# Supplementary material for: Integrating Women and Girls’ Nutrition Services into Health Systems in Low- and Middle-Income Countries: A Systematic Review
Source: Nutrients. 2022 Oct 25;14(21):4488. doi: 10.3390/nu14214488 (PMC9657561; doi:10.3390/nu14214488)
Supplement: Supplementary file 1 [file nutrients-14-04488-s001.zip › nutrients-1929953-supplementary.pdf]

Supplementary Materials

Supplementary Table S1: Search terms

	(women OR female* OR girl* OR adolescen* OR teenager* OR mother* OR maternal OR “reproductive age” OR reproduction OR pregnan* OR pregnant women)
AND	(nutrition OR "Malnutrition"[Mesh] OR undernutrition OR intervention OR “nutrition intervention” OR “nutrition programme” OR “nutrition program” OR “nutrition-specific intervention” OR “nutrition specific intervention” OR “nutrition education” OR “nutrition counselling” OR “maternal nutrition” OR supplement* OR fortifi* OR “food fortification” OR micronutrient* OR “micronutrient intake” OR macronutrient* OR “macronutrient intake” OR anaemi* OR anemi* OR “iron deficiency” OR “iron deficiency anaemia” OR “iron deficiency anemia” OR “iron-folate” OR “iron-folic acid” OR “IFA” OR “vitamin A” OR “vitamin A deficiency” OR “vitamin A supplementation” OR zinc* OR “zinc deficiency” OR “zinc supplementation” OR “calcium supplementation” OR calcium OR “multiple micronutrients” OR “multiple micronutrient supplementation” OR “MMS” OR “MMN” OR “UNIMMAP” OR “balanced energy protein supplementation” OR “dietary diversity” OR “dietary intake” OR “diet intake”)
AND	("Delivery of Health Care, Integrated"[Mesh] OR “health services” OR “health system” OR “health system strengthening” OR “health integration” OR "Reproductive Health Services"[Mesh] OR “reproductive services” OR “reproductive health” OR “delivery of health care” OR “community health workers” OR “CHW” OR "Health Policy"[Mesh] OR "Prenatal Care"[Mesh] OR “antenatal care” OR “postnatal care” OR “ANC” OR “maternal and child survival program” OR “maternal and child survival programme” OR "Maternal Health Services"[Mesh] OR “PMTCT” OR “prevention of mother-to-child transmission” OR “MNCH” OR “maternal neonatal child health” OR “family planning” OR “postpartum family planning”)
AND	(Afghanistan OR Albania OR Algeria OR “American Samoa” OR Angola OR Argentina OR Armenia OR Azerbaijan OR Bangladesh OR Belarus OR Belize OR Benin OR Bhutan OR Bolivia OR “Bosnia and Herzegovina” OR Bosnia OR Herzegovina OR Botswana OR Brazil OR Bulgaria OR “Burkina Faso” OR Burundi OR “Cabo Verde” OR “Cape Verde” OR Cambodia OR Cameroon OR “Central African Republic” OR Chad OR China OR Colombia OR Comoros OR “Democratic Republic of Congo” OR “Republic of Congo” Or Congo OR “Costa Rica” OR “Cote D’Ivoire” OR “Ivory Coast” OR Cuba OR Djibouti OR Dominica OR “Dominican Republic” OR Ecuador OR Egypt OR “El Salvador” OR “Equatorial Guinea” OR Eritrea OR Eswatini OR Swaziland OR Ethiopia OR Fiji OR Gabon OR Gambia OR Georgia OR Ghana OR Grenada OR Guatemala OR Guinea OR “Guinea-Bissau” OR Guyana OR Haiti OR Honduras OR India OR Indonesia OR Iran OR Iraq OR Jamaica OR Jordan OR Kazakhstan OR Kenya OR Kiribati OR Korea OR Kosovo OR Kyrgyzstan OR “Lao PDR” OR Laos OR Lebanon OR Lesotho OR Liberia OR Libya OR Madagascar OR Malawi OR Malaysia OR Maldives OR Mali OR “Marshall Islands” OR Mauritania OR Mexico OR Micronesia OR Moldova OR Mongolia OR Montenegro OR Morocco OR Mozambique OR Myanmar OR Namibia OR Nepal OR Nicaragua OR Niger OR Nigeria OR “North Macedonia” OR Pakistan OR “Papua New Guinea” OR Paraguay OR Peru OR Philippines OR “Russian Federation” OR Rwanda OR Samoa OR “Sao Tome and Principe” OR Senegal OR Serbia OR “Sierra Leone” OR “Solomon Islands” OR Somalia OR “South Africa” OR “South Sudan” OR “Sri Lanka” OR “St. Lucia” OR “St. Vincent and the Grenadines” OR Sudan OR Suriname OR “Syrian Arab Republic” OR Syria OR Tajikistan OR Turkmenistan OR Tuvalu OR Uganda OR Ukraine OR Uzbekistan OR Vanuatu OR Venezuela OR Vietnam OR “West Bank and Gaza” OR “West Bank” OR Gaza OR Yemen OR Yemen OR Zambia OR Zimbabwe)
OR	(“Low and middle income” OR “Low income” OR Middle income” OR “LMIC” OR “LAMI” OR “LAMI countries” OR “Low middle income” OR “Low-middle income” OR “Low-middle-income” OR “Lower middle income” OR “Third world” OR “Developing countries” OR “Developing country” OR “Underdeveloped” OR “Developing or less” OR “Urban” OR “Urban slum” OR “Transitional countries” OR “Transitional country” OR “Global South”)

Supplementary Table S2: "PICO" framework

**P**	Population	Women of reproductive age (including pregnant women), aged 15-49 years (as classified by World Health Organization), living in a low or middle income country (classified by the World Bank).
**I**	Intervention	Any peer reviewed publications that describe the integration of nutrition-specific interventions/services into existing health systems.
**C**	Comparison	**None required**
**O**	Outcome	**None required** - focusing on the extent to which maternal nutrition interventions are integrated, being open to all maternal and infant outcomes.

Supplementary Table S3: Data extraction table, detailing each included article arranged by intervention type

Intervention type	Reference	Country/ Region	Maternal & Child outcome(s) of interest	Target population	Nutrition intervention	Integration	Key conclusions/recommendations
Energy & protein supplementation							
	De Silva et al., 2019 [4]	Asia and the Pacific	- Birth weight (child).<br>- Energy deficiency (maternal).	- Women of reproductive age (WRA).	- Energy & protein supplementation programme.	- Energy & protein supplementation policies have been implemented and aimed at reducing undernutrition and micronutrient deficiencies amongst WRA.<br>- 9 out of 11 countries in the South-East region and 15 out of 26 countries in the Western Pacific region had incorporated goals/targets relating to maternal nutrition services into their national policies.	Advocates for expanding nutrition policies, integrating surveillance data and implementing targeted interventions to improve adolescent health and nutrition outcomes.
	Lassi et al., 2013 [40]	Global	- Neonatal mortality (child).<br>- Small for gestational age (child).<br>- Stillbirth (child).<br>- Birth weight (child).	- WRA.	- Energy & protein supplementation programme.	- Programme was delivered through antenatal care (ANC) services to WRA.	- There is a significant lack of research on the effect of energy and protein supplementation on both child and maternal outcomes.

	Noznesky et al., 2012 [54]	India	- Birth weight (child).<br>- Energy deficiency (maternal).<br>- Underweight (maternal).<br>- Anaemia (maternal).<br>- Birth weight (child).	- Pregnant and lactating women.<br>- Pregnant adolescent girls.	- Energy & protein supplementation programme.	- Energy & protein supplementation is delivered through the Indian Primary Healthcare System, the Integrated Child Development Services, secondary schools and the Targeted Public Distribution System.	- Advocates for interventions targeting non-pregnant, non-lactating adolescents or WRA as currently these subgroups are not being targeting and this hinders the efforts to improve maternal nutrition across the region.<br>- Advocates for strengthening policies and programs encouraging WRA to postpone first pregnancy until the mother is at least 18 years old to improve health outcomes of both mother and child.
Food fortification							
	Barker et al., 2018 [10]	Global	- Birth weight (child).<br>- Body mass index (BMI) (maternal).<br>- Stillbirth (child).<br>- Neonatal mortality (child).	- WRA.	- Iodised salt fortification programme.<br>- Folic acid fortification programme.	- Observed reduction in neural tube defects in USA, Canada, Chile, Costa Rica and South Africa where folic acid fortification was made mandatory.<br>- Low- and middle-income countries (LMICs) provided political support for the adoption and integration of pre-conception fortification strategies to address determinants of maternal malnutrition.	- Author states that pre-conception interventions need to be supported by political will in order to be effective in achieving health growth trajectories in future generations.

	Mason et al., 2012 [45]	Ethiopia, India, Nigeria, Global	- Birth weight (child).<br>- Neonatal growth (child).<br>- Morbidity (maternal).<br>- Mortality (maternal).<br>- Anaemia (maternal).<br>- Iodine deficiency (maternal).<br>- Vitamins A,B,C,D deficiencies (maternal).	- WRA.<br>- Pregnant and lactating women.	- Vitamin A, B, C, calcium, iron, and zinc fortified wheat flour programme.<br>- Iron-fortified wheat flour.	- Fortified flour is provided for pregnant and lactating women and distributed through the Integrated Child Development services in India.<br>- State policy in India exists regarding the supply of fortified foods and distribution through the Targeted Public Distribution System.<br>- In Nigeria and Ethiopia, national guidelines and initiatives exist regarding food fortification of iron-fortified wheat flour and iodised salt.	- Awareness of the importance of maternal nutrition was observed as a limiting factors when identifying how well integrated maternal nutrition services were.<br>- There is adequate support and funding for maternal nutrition services and is often low priority; author calls for a information campaign to raise awareness of its importance.
	Mgamb et al., 2017 [47]	Kenya	- Folate deficiency (maternal).<br>- Knowledge of folic acid fortified flour (maternal).	- Pregnant women.	- Folic acid fortified flour programme.	- Programme was integrated into ANC services at a maternity hospital.<br>- Government of Kenya made fortification of maize and wheat flour with folic acid mandatory in 2012.	
	Nguyen et al., 2020 [53]	Southeast Asia	- Birth weight (child).<br>- Stunting (child).<br>- Wasting (child).<br>- Underweight (child).<br>- Overweight (child).	- WRA.	- Vitamin A and iron food fortification programme.	- Food fortification interventions were listed as one of the national nutrition strategies (NNS) in 8 out of the 11 countries within Southeast Asia; these countries also	- Advocates for updating information regarding nutrition strategies and indicators in national databases in order to facilitate cross-checking and comparing data across countries within the region;

			- Iron deficiency anaemia (child).<br>- Vitamin A deficiency (child).<br>- Iron deficiency anaemia (maternal).<br>- Underweight (maternal).<br>- Overweight & obesity (maternal).			listed relevant policies associated with regulation of food fortification.	this would also help coordinate well-designed and measured nutrition policies.
	Victora et al., 2012 [79]	Sub-Saharan Africa, South Asia, Global	- Birth weight (child).<br>- Iron deficiency anaemia (child)	- WRA.<br>- Pregnant and lactating women.	- Iron & folic acid wheat-flour fortification programme.	- 36 countries in Sub-Saharan Africa have universal and routine salt iodisation.<br>- Globally, national implementation and coverage of iron & folic acid wheat-flour fortification increased post-integration; from 18% in 2004 to 27% in 2007.	- Author states that most food fortification programmes are directed at children and their outcomes rather than pregnant women; this means there is a lack of data around maternal outcomes such as gestational weight gain, nutritional intake during pregnancy etc.<br>- Advocates for greater integration into antenatal clinics and a focus on maternal nutrition outcomes.
Nutrition education/counselling							
	Barker et al., 2019 [11]	Sub-Saharan Africa	- Breastfeeding practices (maternal).<br>- Undernourishment (maternal).<br>- Undernourishment (child).	- Pregnant women living with HIV.	- Nutrition counselling (including breastfeeding practices).	- Programme was scaled-up and integrated into perinatal and antenatal services as part of the Partnership for HIV-free Survival initiative, with the assistance of Ministry of Health departments across Tanzania and Uganda.	- Further integration is needed into government programmes and better sustainability of quality insurance capability throughout health systems.

						- Kenya, Mozambique and Lesotho delivered the programme, however there was less engagement and no plans to scale-up.	
	Chakrabarti et al., 2019 [16]	India	- Educational level (maternal).<br>- Stunting (child).	- Pregnant and lactating women.	- Nutrition education programme.	- Programme is delivered through the Integrated Child Development services.<br>- India's policy reforms have improved coverage of the programme at a national level and managed to reach marginalised groups.	- Author states the programme needs more effective implementation to reach women from low educational and schooling backgrounds, as well as women from lower socioeconomic class.
	De Silva et al., 2019 [4]	Asia and the Pacific	- Birth weight (child).<br>- Energy deficiency (maternal).	- WRA.	- Nutrition education and counselling programme.	- 9 out of 11 countries in the South-East region and 15 out of 26 countries in the Western Pacific region had incorporated goals/targets relating to maternal nutrition services into their national policies.	- Innovations to improve delivery of nutrition education should include social media and other networks to communicate messages on a larger scale.
	Levin et al., 2019 [41]	Kenya	- Vitamin A deficiency (maternal).	- Pregnant women.	- Nutrition education and agricultural programme (promoting the production and consumption of orange-fleshed sweet potato)	- Programme is integrated into the USAID/Kenya AIDs, Population and Health Integrated Assistance Program and combined with antenatal support and nutrition/Infant and young child feeding (IYCF) education.	

	Mason et al., 2012 [45]	Ethiopia, India, Nigeria, Global	- Birth weight (child).<br>- Neonatal growth (child).<br>- Morbidity (maternal).<br>- Mortality (maternal).<br>- Anaemia (maternal).<br>- Iodine deficiency (maternal).<br>- Vitamins A,B,C,D deficiencies (maternal).	- WRA.<br>- Pregnant and lactating women.<br>- Adolescent girls (11-18 years old).	- Nutrition education and counselling programme.	- Nutrition education and counselling was delivered in schools through the Integrated Child Development services in India.	- Awareness of the importance of maternal nutrition was observed as a limiting factors when identifying how well integrated maternal nutrition services were.<br>- There is adequate support and funding for maternal nutrition services and is often low priority; author calls for a information campaign to raise awareness of its importance.
	Muehlhoff et al., 2017 [49]	Cambodia, Malawi	- Vitamin A deficiency (maternal).<br>- Energy intake (child, maternal).<br>- Protein intake (child, maternal).<br>- Anaemia (maternal).	- WRA.	- Nutrition education (including IYCF-practices and cooking sessions).	- Programme follows Food and agriculture organisation's (FAO) integrated agriculture-IYCF nutrition education approach to promote behaviour change and aimed at improving dietary quality.<br>- Programme is facilitated by the National Nutrition Programme, Provincial Department of Health, Provincial Department of Women's Affairs and non-governmental organisations (NGOs).<br>- The governments of both countries trained volunteers as	- Emphasises the importance of both household and community level action in the support of successful and integration, accessibility and coverage.

						Community Nutrition Promoters to conduct nutrition education activities at village level.	
	Nguyen et al., 2017 [52]	Banglades h	- Dietary diversity (maternal).<br>- Breastfeeding practices (maternal).<br>- Micronutrient supplement intake (maternal).	- Pregnant and lactating women.	- Nutrition counselling programme (including lactation and breastfeeding practices).	- The Alive & Thrive initiative integrated this programme into the existing Maternal, Neonatal, and Child Health (MNCH) programme in Bangladesh.<br>- Delivered through ANC services as part of a multiple nutrition-focused MNCH approach.	- Programme was effective when facilitated by a strong system for early pregnancy detection and ANC delivery.<br>- Successful implementation and impact requires strengthening of health systems and building a network of skilled frontline workers.
	Noznesky et al., 2012 [54]	India	- Birth weight (child).<br>- Energy deficiency (maternal).<br>- Underweight (maternal).<br>- Anaemia (maternal).<br>- Birth weight (child).	- WRA.<br>- Adolescent girls in school.	- Nutrition education programme.	- Programme was delivered to WRA in local Anganwadi centres and to adolescent girls in schools by Community Health Workers (CHWs).<br>- Integrated into the School Anaemia Control Programme.	

	Riang'a et al., 2020 [59]	Kenya	- Anaemia (maternal).<br>- Mortality (child).	- WRA.<br>- Pregnant and lactating women.	- Nutrition counselling programme (including advice on an adequate and nutritious diet and food-related problems during pregnancy)	- Programme was created following government policy guidelines to strengthen maternal nutrition assessment and improve access to nutrition education and knowledge.<br>- Programme was integrated into ANC services and delivered in government health facilities.	- Adherence to the programme was affected by late initial booking to ANC services; more needs to be done to promote and encourage pregnant women to book appointments as early as they can in order to enrol in the integrated supplementation programme.
	Robert et al., 2017 [60]	Peru	- Iron-deficiency (child).<br>- Anaemia (child).<br>- Anthropometric measurements (child).	- Mothers.	- Nutrition counselling programme (including advice on IYCF practices and nutritional value of local market foods).	- Programme was delivered through government health facilities, ranging from small health centres to large maternity centres.<br>- Nutrition counselling was offered as part of the medical consultation with mothers and their children.	- To promote healthy growth in children there needs to be more nutrition-specific interventions that focus on delivering via health worker-caregiver interactions.
	Ruton et al., 2018 [62]	Rwanda	- Anthropometric measurements (child).<br>- Malnutrition prevalence (child).	- Pregnant and lactating women.<br>- Mothers.	- Nutrition counselling mobile health service.	- Programme was scaled-up and delivered nationally by the Rwandan government.<br>- Mothers were given mobile phones to report maternal and child indicators which would link them with maternal care services.	- The mobile health system was effective in increasing the usage of maternal and child health services however it would be even more effective if combined as a package intervention.

	Salam et al., 2016 [64]	Pakistan	- Pre-eclampsia (maternal).	- WRA.<br>- Pregnant and lactating women.<br>- Mothers.	- Nutrition counselling programme.	- Programme is delivered across two districts in Pakistan at community level and health workers are first point of contact for mothers/WRA.<br>- Government facilities provide support for delivery of the programme and referrals.<br>- Women are referred into maternal health services and ANC clinics from this point.	
	Saldanha et al., 2012 [65]	Ethiopia	- Anaemia (maternal).<br>- Thinness (maternal).<br>- Stunting (maternal).	- Mothers.<br>- Pregnant and lactating women.	- Nutrition education and counselling programme.	- Programme was integrated into ANC services and community health workers were employed to mobilise women to attend ANC.<br>- Health extension programme was scaled-up to improve pregnant and lactating women in their access to health services.	
	Saronga et al., 2019 [66]	India, Indonesia, Kenya	- Adherence to taking iron-folic acid (IFA) supplements (maternal).<br>- % pregnant women who received micronutrient supplements	- Pregnant and lactating women.	- Nutrition education and counselling mobile health service.	- Programme involved texts/audio messages sent to mothers about important aspects of ANC, reminders to collect their IFA supplements and other health and nutrition advice as well as diet counselling.	

			(maternal).<br>- % pregnant women who received dietary counselling (maternal).<br>- Birthweight (child).<br>- Haemoglobin level (maternal).<br>- Neonatal mortality (child).			- Programme was delivered as part of maternal health services and integrated into ANC services.	
	Sethi et al., 2019 [68]	India	- Gestational weight gain (maternal).<br>- Birthweight (child).<br>- Haemoglobin levels (maternal).	- Pregnant and lactating women.<br>- Mothers.	- Nutrition counselling programme.	- State government launched this programme in order to bundle together various nutrition interventions (spot feeding hot meals, heath services, nutrition counselling, etc.) together and deliver to women throughout pregnancy and up tp 6 months post-partum.	- Author states that this scheme potentially could be very efficient and cost-effective in providing a platform for multiple nutrition-specific and nutrition-sensitive interventions to improve maternal and child health outcomes.
	Torlesse et al., 2021 [74]	Afghanistan, Bangladesh, Nepal, India, Pakistan	- Meal frequency (maternal).<br>- Adherence to taking IFA and multiple micronutrient supplements (maternal).<br>- Consumption of nutritious and diverse foods (maternal).<br>- Quantity of food intake (maternal).	- WRA.<br>- Pregnant and lactating women.	- Nutrition education and counselling (including IYCF practices).	- Programme was integrated into ANC services and counselling was provided at health facility, community, household and individual levels.	- Author states that inequalities in access to nutrition services as well as limited investment into training for health workers constrains the quality of counselling.<br>- More needs to be done to integrate counselling into M&E mechanisms in order to assess where improvements are needed.

	Varghese et al., 2014 [77]	India	- Neonatal mortality (child).<br>- Breastfeeding initiation within 5 hours postpartum (maternal).	- Pregnant and lactating women.<br>- Mothers.	- Nutrition counselling programme.	- Programme was launched as part of a plan to improve maternal and neonatal care and integrated into various health facilities (mainly hospitals) across India.<br>- Healthworkers, called "Yashodas" were places at hospitals and health facilities to provide support and care for mothers during their stay; this included nutrition counselling, breastfeeding counselling and family planning advice.	
	Victora et al., 2012 [79]	Sub-Saharan Africa, South Asia, Global	- Birth weight (child).<br>- Iron deficiency anaemia (child)	- WRA.<br>- Pregnant and lactating women.	- Nutrition education and counselling programme.	- Community nutrition centres targeted WRA and delivered the programme to them in India.<br>- In Madagascar, the programme was delivered via local NGOs and with the help of community nutrition workers.	- Advocates for greater integration into antenatal clinics and a focus on maternal nutrition outcomes.
Home food distribution							
	Chakrabarti et al., 2019 [16]	India	- Educational level (maternal).<br>- Stunting (child).	- Pregnant and lactating women.	- Food supplementation programme.	- Programme is delivered through the Intergrated Child Development services.<br>- India's policy reforms have improved coverage of the	- Author states the programme needs more effective implementation to reach women from low educational and schooling backgrounds, as well as

						programme at a national level and managed to reach marginalised groups.	women from lower socioeconomic class.
Micronutrient supplementation							
	Abdullahi et al., 2014 [42]	Sudan	- Birth weight (child).<br>- Haemoglobin level (maternal).<br>- Anaemia (maternal).	- Pregnant women.	- IFA supplementation programme.	- Programme was integrated into ANC services and into hospitals.<br>- Government of Sudan have established policies recommending folic acid supplementation for pregnant women in the first trimester and combines IFA supplements in the second and third trimester.	
	Arega Sadore et al., 2015 [36]	Ethiopia	- Adherence to taking IFA supplements (maternal).<br>- Knowledge of anaemia (maternal).	- Pregnant women.	- IFA supplementation programme.	- Programme was integrated into ANC services and into seven health centres and thirty five health posts within the community.	- Author concluded that health workers promoting mothers to attend ANC at least 4 times could improve compliance to IFA supplementation programmes; more effort needs to be directed towards mobilising community health workers in this area to improve maternal health outcomes.
	Assefa et al., 2019 [76]	Ethiopia	- Adherence to taking IFA supplements (maternal).<br>- Knowledge of anaemia	- Pregnant women.<br>- WRA.	- IFA supplementation programme.	- Programme was integrated into ANC service which were delivered through the main hospital in the	- Author stated that efforts to strengthen health care facilities abilities to provide information and create awareness of all-cause anaemia and the importance

			(maternal).<br>- Knowledge of the benefits of supplementation (maternal).			town and town health centres.	of IFA supplementations during pregnancy.
	Bannink et al., 2015 [9]	Uganda	- Adherence to taking folic acid supplements (maternal).<br>- Knowledge of spina bifida (maternal).<br>- Knowledge of the importance of folic acid (maternal).	- Pregnant women.<br>- WRA.	- Folic acid supplementation programme.	- Programme was integrated into ANC services and delivered by health workers.	- Women attending health education at the health facilities were more likely to adhere to folic acid supplementation programmes; more emphasis needs to be on integrating health education into supplementation programmes and ANC.
	Berti et al., 2018 [12]	Central America, Southeast Asia, South Asia, Sub-Saharan Africa	- Anaemia (maternal).<br>- Iron status (maternal).<br>- Neonatal outcomes (child).	- Pregnant women.<br>- WRA.	- Multiple micronutrient supplementation programme.	- Nicaragua: implementation of MMN is part of the national Integrated Anaemia Control Strategy, which is part of the National Micronutrient Plan.<br>- Nepal: implementation of IFA supplementation as part of the Iron Intensification Project within Nepal's National Plan for Action for the Control of Anaemia. Government of Nepal have strong partnerships with NGOs in supporting IFA supplementation integration into ANC.<br>- Vietnam:	- Implementation and integration strategies were most effective in programmes that were facilitated by continued promotional and education activities, and that were context-specific.

						implementation of IFA as part of the national Iron-deficiency Anaemia Control Programme includes daily universal supplementation of WRA.	
	Birhanu et al., 2018 [13]	Ethiopia	- Adherence to taking IFA supplements (maternal).<br>- History of anaemia (maternal).	- Pregnant women.	- IFA supplementation programme.	- Programme was integrated into ANC services and delivered in a hospital.	
	Chikakuda et al., 2018 [17]	Malawi	- Anaemia (maternal).<br>- Haemoglobin levels (maternal).<br>- Serum folate levels (maternal).<br>- Gestational weight gain (maternal).<br>- Birth weight (child).<br>- Adherence to taking IFA supplements (maternal).	- Pregnant women.	- IFA supplementation programme.	- Programme was integrated into ANC services and delivered in a hospital.	- Author stated that compliance to IFA supplementation programmes could be improved if the government of Malawi improves the supply of IFA tablets to ANC clinics.

	De Silva et al., 2019 [4]	Asia and the Pacific	- Birth weight (child).<br>- Anaemia (maternal).	- Pregnant women.<br>- WRA.	- IFA supplementation programme.	- IFA supplementation policies have been implemented and aimed at reducing micronutrient deficiencies amongst WRA.<br>- 9 out of 11 countries in the South-East region and 15 out of 26 countries in the Western Pacific region had incorporated goals/targets relating to maternal nutrition services into their national policies.	- Advocates for expanding nutrition policies, integrating surveillance data and implementing targeted interventions to improve adolescent health and nutrition outcomes.
	Desta et al., 2019 [19]	Ethiopia	- Adherence to taking IFA supplements (maternal).<br>- Anaemia (maternal).<br>- Knowledge of IFA supplementation and anaemia (maternal).	- Pregnant women.	- IFA supplementation programme.	- Programme was integrated into ANC services.	- Adherence to IFA supplementation programmes was improved when women received counselling and education alongside.
	Dubik et al., 2019 [21]	Ghana	- Adherence to taking IFA supplements (maternal).<br>- Anaemia (maternal).<br>- Knowledge of anaemia (maternal).	- Adolescent girls.	- IFA supplementation programme.	- Programme was developed by Ghana's Ministry of Health and was integrated into junior high schools for all adolescent girls.	- Educating adolescent girls on anaemia and the benefits of IFA would improve compliance to the programme.

	Feldhaus et al., 2016 [24]	Nepal	- Pre-eclampsia (maternal).<br>- Adherence to taking calcium supplements (maternal).<br>- Hypertension (maternal).	- Pregnant women.	- Calcium supplementation programme.	- Programme was integrated into ANC services and delivered through health facilities that were provided by the District Health Office, and supported by community health workers.	- Author stated that more research is needed to define ways to improve early initiation of women into ANC.
	Gebreamlak et al., 2017 [26]	Ethiopia	- Adherence to taking IFA supplements (maternal).	- Pregnant women.	- IFA supplementation programme.	- Programme was integrated into ANC services.	
	Gebremariam et al., 2017 [20]	Ethiopia	- Adherence to taking IFA supplements (maternal).<br>- Anaemia (maternal).<br>- Knowledge of IFA supplementation and anaemia (maternal).	- Pregnant women.	- IFA supplementation programme.	- Programme was integrated into ANC services and delivered in a hospital setting.	- Adherence to IFA supplementation programmes was improved when women received counselling from health workers.
	Gebremichael et al., 2019 [27]	Ethiopia	- Adherence to taking IFA supplements (maternal).<br>- Knowledge of anaemia (maternal).	- Pregnant women.<br>- WRA.	- IFA supplementation programme.	- Programme was integrated into ANC services and delivered in a hospital.	
	Gebremichael et al., 2020 [28]	Ethiopia	- Adherence to taking IFA supplements (maternal).	- Pregnant women.<br>- WRA.	- IFA supplementation programme.	- Programme was integrated into ANC services and delivered through government health institutions.	- Efforts should be made to raise awareness of IFA supplementation amongst pregnant women and WRA.<br>- Nutrition counselling should be integrated into IFA

							programmes to improve adherence.
	Jaiswal et al., 2015 [32]	India	- Urinary iodine concentration (maternal).<br>- Thyroid volume (maternal).	- Mothers.<br>- Pregnant women.	- Multi-micronutrient powder supplementation programme and Iodine-containing supplementation programme.	- Programme was integrated into ANC services in an urban tertiary-care hospital.	
	Kamau et al., 2020 [34]	Kenya	- Adherence to taking IFA supplements (maternal).<br>- Knowledge of IFA supplements (maternal).<br>- Anaemia (maternal).	- Pregnant women.	- IFA supplementation programme.	- Programme was integrated into ANC services and delivered in five public health facilities.<br>- CHWs distributed the supplements (this is the first time distribution via CHWs has been carried out in Kenya).	- The role of CHWs in community-based approach to supplementation distribution and IFA education is not defined clearly in national policy; the success of this programme suggests it should be.
	Kim et al., 2017 [37]	India	- Neonatal mortality (child).<br>- Mortality (maternal).	- WRA.<br>- Pregnant women.	- IFA supplementation and vitamin A supplementation programme.	- Programme was delivered through two government services: the Integrated Child Development services and National Rural Health Mission in India.	- Though the programme is delivered through government health services, there is a lack of convergence with health and nutrition policy at state level.
	Kiwanuka et al., 2017 [39]	Uganda	- Adherence to taking IFA supplements (maternal).	- Pregnant women.	- Iron supplementation programme.	- Programme was integrated into ANC services and delivered in a hospital.	
	Lassi et al., 2013 [40]	Global	- Neural tube defects (child).<br>- Miscarriage (maternal).<br>- Pre-term	- WRA.	- Folic acid supplementation, IFA supplementation, MMN	- Cochrane review of various maternal nutrition programmes integrated into policy globally; interventions	- Author calls for these interventions to be scaled-up and highlights that policy and national regulations are essential in improving

			delivery (maternal).<br>- Adherence to taking folic acid supplements (maternal).<br>- Gestational weight (maternal).<br>- Pre-pregnancy underweight and overweight (maternal).<br>- Iron-deficiency anaemia (maternal).<br>- Birth weight (child).<br>- Stillbirth (child).<br>- Hypertension (maternal).<br>- Pre-eclampsia (maternal).		supplementation, Calcium supplementation, programme.	were grouped into proposed packages of care delivery through public health services (at community, health centre and hospital levels.)	implementation and coverage.
	Mason et al., 2012 [45]	Ethiopia, India, Nigeria, Global	- Birth weight (child).<br>- Neonatal growth (child).<br>- Morbidity (maternal).<br>- Mortality (maternal).<br>- Anaemia (maternal).<br>- Iodine deficiency (maternal).<br>- Vitamins A,B,C,D deficiencies (maternal).	- WRA.<br>- Pregnant and lactating women.<br>- Adolescent girls (11-18 years old).	- Iron-folic acid (IFA) supplementation programme.	- Iron-folic acid supplements were delivered in schools through the Integrated Child Development services in India.<br>- Plans across India to scale-up this service and supplement adolescent girls weekly.	- The policies for IFA supplementation all exist in India, however there is a need for more resources in order to increase accessibility and coverage.

	Mason et al., 2012 [45]	Ethiopia, India, Nigeria, Global	- Birth weight (child).<br>- Neonatal growth (child).<br>- Morbidity (maternal).<br>- Mortality (maternal).<br>- Anaemia (maternal).<br>- Iodine deficiency (maternal).<br>- Vitamins A,B,C,D deficiencies (maternal).	- WRA.<br>- Pregnant and lactating women.	- IFA and MMN supplementation programme.	- In Nigeria, local NGOs work in partnership with the government to deliver supplements to women and children as part of the National Integrated Maternal and Child Health campaigns.<br>- Overall, MMN and IFA programmes were integrated into community-based nutrition services and ANC services.	- Awareness of the importance of maternal nutrition was observed as a limiting factors when identifying how well integrated maternal nutrition services were.<br>- There is adequate support and funding for maternal nutrition services and is often low priority; author calls for a information campaign to raise awareness of its importance.
	Mistry et al., 2018 [48]	India	- Anaemia (maternal).<br>- Iron-deficiency anaemia (maternal).	- Pregnant women.	- Iron supplementation programme.	- Programme was integrated into ANC services in government health centres.	- Good systems in place for tackling anaemia however policy-makers are interested in integrating antenatal tobacco screening for anaemia control into these services.
	Nguyen et al., 2017 [52]	Banglades h	- Dietary diversity (maternal).<br>- Breastfeeding practices (maternal).<br>- Micronutrient supplement intake (maternal).	- Pregnant and lactating women.	- IFA and calcium supplementation programme.	- The Alive & Thrive initiative integrated this programme into the existing Maternal, Neonatal, and Child Health (MNCH) programme in Bangladesh.<br>- Delivered through ANC services as part of a multiple nutrition-focused MNCH approach.	- Programme was effective when facilitated by a strong system for early pregnancy detection and ANC delivery.<br>- Successful implementation and impact requires strengthening of health systems and building a network of skilled frontline workers.

	Nguyen et al., 2019 [83]	India	- Dietary diversity (maternal).<br>- Adherence to taking calcium supplements (maternal).<br>- Adherence to taking IFA supplements (maternal).<br>- Gestational weight (maternal).	- Pregnant women.	- Iron supplementation and calcium supplementation programme.	- Programmes were integrated into child and maternal health services and delivered as part of the government Integrated Child Development Scheme and Reproductive, Maternal, Newborn, Child and Adolescent health programmes.<br>- Strong policy commitments are in place to improve maternal nutrition.	- Despite strong policy recommendations for maternal nutrition, these services are not reaching pregnant women and coverage is low.
	Nguyen et al., 2020 [53]	Southeast Asia	- Birth weight (child).<br>- Stunting (child).<br>- Wasting (child).<br>- Underweight (child).<br>- Overweight (child).<br>- Iron deficiency anaemia (child).<br>- Vitamin A deficiency (child).<br>- Iron deficiency anaemia (maternal).<br>- Underweight (maternal).<br>- Overweight & obesity (maternal).	- WRA.	- MMN supplementation programme.	- MMN supplementation programmes were listed as one of the national nutrition strategies (NNS) in 9 out of the 11 countries within Southeast Asia; these countries also listed relevant policies associated with regulation of MMN supplementation for pregnant women.	- Advocates for updating information regarding nutrition strategies and indicators in national databases in order to facilitate cross-checking and comparing data across countries within the region; this would also help coordinate well-designed and measured nutrition policies.

	Noznesky et al., 2012 [54]	India	- Birth weight (child).<br>- Energy deficiency (maternal).<br>- Underweight (maternal).<br>- Anaemia (maternal).<br>- Birth weight (child).	- WRA.<br>- Adolescent girls in school.	- IFA supplementation programme.	- Programme was delivered as part of the government of Bihar's strategy to reduce maternal undernutrition by implementing several interventions aimed at improving maternal and birth outcomes.<br>- Integrated into the School Anaemia Control Programme.	- Advocates for the distribution of packages with products and information promoting good reproductive health and good nutrition for newly married couples who would benefit from being targeted by this programme.
	Omotayo et al., 2018 [55]	Kenya	- Pre-eclampsia (maternal).<br>- Anaemia (maternal).<br>- Adherence to taking calcium supplements (maternal).<br>- Adherence to taking IFA supplements (maternal).	- Pregnant women.	- Calcium supplementation and IFA supplementation programme.	- Programme was integrated into primary healthcare facilities in Kenya.	- Programme is yet to be integrated specifically into ANC services.
	Ouedraogo et al., 2019 [56]	Niger	- Adherence to taking IFA supplements (maternal).<br>- Gestational weight (maternal).<br>- Anaemia (maternal).	- Pregnant women.	- IFA supplementation programme.	- Programme was integrated into ANC services and selected integrated health centres.	

	Paudyal et al., 2021 [57]	Nepal	- Anaemia (maternal).	- Pregnant women.<br>- Mothers.	- IFA supplementation programme.	- Government of Nepal launched the Iron Intensification programme where CHWs promoted and distributed supplements at ANC clinics.	
	Riang'a et al., 2020 [59]	Kenya	- Anaemia (maternal).<br>- Mortality (child).	- WRA.<br>- Pregnant and lactating women.	- IFA supplementation programme.	- Programme was created following government policy guidelines to address maternal anaemia amongst WRA.<br>- Programme was integrated into ANC services and delivered in government health facilities.	- Adherence to the programme was affected by late initial booking to ANC services; more needs to be done to promote and encourage pregnant women to book appointments as early as they can in order to enrol in the integrated supplementation programme.
	Roche et al., 2018 [61]	Indonesia	- Anaemia (maternal).	- Adolescent girls.<br>- WRA.	- IFA supplementation programme.	- Indonesia's Ministry of Health amended its national programme for prevention of anaemia in WRA and adolescent girls to align with WHO guidance and the programme is delivered through the existing school health programme (names UKS/M).	

	Saldanha et al., 2012 [65]	Ethiopia	- Anaemia (maternal).<br>- Thinness (maternal).<br>- Stunting (maternal).	- Mothers.<br>- Pregnant and lactating women.	- IFA supplementation programme.	- Programme was integrated into ANC services and community health workers were employed to mobilise women to attend ANC.<br>- Health extension programme was scaled-up to improve pregnant and lactating women in their access to health services.	
	Salam et al., 2016 [64]	Pakistan	- Pre-eclampsia (maternal).	- WRA.<br>- Pregnant and lactating women.<br>- Mothers.	- IFA supplementation programme.	- Programme is delivered across two districts in Pakistan at community level and health workers are first point of contact for mothers/WRA.<br>- Government facilities provide support for delivery of the programme and referrals.<br>- Women are referred into maternal health services and ANC clinics from this point.	
	Sedlander et al., 2020 [67]	India	- Anaemia (maternal).<br>- Adherence to taking IFA supplements (maternal).	- WRA.<br>- Pregnant women.	- IFA supplementation programme.	- Government of India recommends daily IFA supplementation for pregnant women and weekly supplementation for WRA.<br>- IFA supplements are free and distributed by the government.	

	Sethi et al., 2019 [68]	India	- Gestational weight gain (maternal).<br>- Birthweight (child).<br>- Haemoglobin levels (maternal).	- Pregnant and lactating women.<br>- Mothers.	- Calcium supplementation and IFA supplementation programme.	- State government launched this programme in order to bundle together various nutrition interventions (spot feeding hot meals, heath services, nutrition counselling, etc.) together and deliver to women throughout pregnancy and up tp 6 months post-partum.	- Author states that this scheme potentially could be very efficient and cost-effective in providing a platform for multiple nutrition-specific and nutrition-sensitive interventions to improve maternal and child health outcomes.
	Soekarjo et al., 2018 [70]	Indonesia	- Anaemia (maternal).	- Adolescent girls.	- IFA supplementation programme.	- There is only one nutrition-specific policy and programme that is designed to improve adolescent nutritional status; the IFA supplementation programme has been integrated into the Guideline for the Prevention and Management of Anaemia among Adolescent Girls and WRA.	- There is a lack of policy and programmes to improve adolescent girls' nutritional status.
	Thapa et al., 2016 [73]	Nepal	- Pre-eclampsia (maternal).<br>- Gestational age (child).<br>- Adherence to taking calcium supplements (maternal).	- Pregnant women.	- Calcium supplementation programme.	- Programme was integrated into ANC services and delivered via CHWs.	

	Varghese et al., 2019 [78]	India	- Iron-deficiency anaemia (maternal).	- Pregnant women.	- IFA supplementation programme.	- Programme was integrated into ANC services and delivered via health workers in primary, secondary and tertiary levels of health care	
	Victora et al., 2012 [79]	Sub-Saharan Africa, South Asia, Global	- Birth weight (child).<br>- Iron deficiency anaemia (child)	- WRA.<br>- Pregnant and lactating women.	- Iron supplementation, IFA supplementation and MMN supplementation programme.	- Community nutrition centres targeted WRA and delivered the programme to them in India.<br>- In Madagascar, the programme was delivered via local NGOs and with the help of community nutrition workers.<br>- IFA supplementation programmes integrated into ANC services were standard component in most, if not all of the countries.	- Advocates for greater integration into antenatal clinics and a focus on maternal nutrition outcomes.
	Wadhwa et al., 2018 [81]	India	- Anaemia (maternal).	- Adolescent girls.	- IFA supplementation programme.	- Programme has been integrated into adolescent-friendly health clinics.<br>- National Adolescent Reproductive and Sexual Health Policy (2006) involves nutrition-specific policies for adolescent girls.	

Delivery platform	Reference	Country/ Region	Maternal & Child outcome(s) of interest	Target population	Nutrition intervention	Integration	Key conclusions/recommendations
Community health worker programmes							
	Altobelli et al., 2017 [25]	Afghanistan, Peru	- Mortality (child).<br>- Morbidity (child).<br>- Breastfeeding practices (maternal).<br>- Growth & anthropometric measurements (child).<br>- Nutrition knowledge (maternal).	- Mothers	- Nutrition counselling programme.	- CHWs recognised as key in closing the gap between health services and communities in need, therefore this programme was scaled-up in government systems and primary health care setting.<br>- "Sharing Histories" is a strategy was developed in partnership with the Afghan Ministry of Health which trains CHWs in sharing health and nutrition knowledge effectively.<br>- Programme used the "Sharing Histories" method to improve CHWs performance in aiding mother's behaviour change towards improving MNCH outcomes.	- "Sharing Histories" is an effective method for improving CHWs ability to communicate health knowledge effectively and can easily be incorporated into CHW programs anywhere.<br>- Policies and guidelines are still needed to support CHWs.

	Amoah et al., 2016 [18]	Ghana	- Morbidity (maternal).	- Pregnant women.	- Mobile health service.	- Programme was delivered via CHWs and encouraged ANC attendance.<br>- CHWs were given mobile phones and would input pregnancy data given by mothers; mothers would then report maternal indicators (such as bleeding, abdominal pain, etc.) and CHWs would be instructed on what to advise the pregnant women and would then link them with ANC services.	
	Noznesky et al., 2012 [54]	India	- Birth weight (child).<br>- Energy deficiency (maternal).<br>- Underweight (maternal).<br>- Anaemia (maternal).<br>- Birth weight (child).	- WRA.<br>- Adolescent girls in school.	- IFA supplementation, Energy-protein supplementation and Nutrition education programmes.	- CHWs helped to create awareness and increase demand for the programme and services.<br>- CHWs including Lady health visitors, Male health workers and Auxiliary nurse midwives constituted the primary healthcare workforce at the community level.<br>- Programme was delivered as part of the government of Bihar's strategy to reduce maternal undernutrition by implementing several interventions aimed at improving	- Advocates for increased support and supervision of CHWs to improve training, problem-solving abilities and flow of information.

						maternal and birth outcomes.<br>- Integrated into the School Anaemia Control Programme.	
	Ruton et al., 2018 [62]	Rwanda	- Anthropometric measurements (child).<br>- Malnutrition prevalence (child).<br>- Mortality (maternal).<br>- Mortality (child).	- Pregnant and lactating women.<br>- Mothers.	- Nutrition counselling mobile health service.	- Programme was scaled-up and delivered nationally via CHWs deployed by the Rwandan government.<br>- Mothers were given mobile phones and health/nutrition messages were communicated to them by CHWs. Mothers would then report maternal and child indicators and CHWs would link them with maternal care services.	- The mobile health system was effective in increasing the usage of maternal and child health services however it would be even more effective if combined as a package intervention.
	Salam et al., 2016 [64]	Pakistan	- Pre-eclampsia (maternal).	- WRA.<br>- Pregnant and lactating women.<br>- Mothers.	- Nutrition counselling programme.	- Programme is delivered across two districts in Pakistan at community level and health workers are first point of contact for mothers/WRA.<br>- Government facilities provide support for delivery of the programme and referrals.<br>- Women are referred into maternal health services and ANC clinics from this point.	

	Stansert Katzen et al., 2020 [71]	South Africa	- Exclusive breastfeeding practices (maternal).<br>- Growth & Anthropometric measurements (child).<br>- Birth weight (child).<br>- Stunting (child).<br>- Wasting (child).	- Pregnant and lactating women.<br>- Mothers.	- CHW home-visit programme.	- CHWs would conduct home visits with mothers and repeatedly assess children for 2 years post-birth.<br>- Programme was delivered through government health facilities in a hospital setting where CHWs recruited new mothers for the programme.	
	Thapa et al., 2016 [73]	Nepal	- Pre-eclampsia (maternal).<br>- Gestational age (child).<br>- Adherence to taking calcium supplements (maternal).	- Pregnant women.	- Calcium supplementation and counselling programme.	- Programme was integrated into ANC services and delivered via CHWs.<br>- CHWs in the form of Female community health volunteers were trained to distribute calcium supplements and counsel pregnant women about calcium use by reinforcing calcium-related health messages.	
Adolescent health services							
	Noznesky et al., 2012 [54]	India	- Birth weight (child).<br>- Energy deficiency (maternal).<br>- Underweight (maternal).<br>- Anaemia (maternal).	- WRA.<br>- Adolescent girls in school.	- IFA supplementation and deworming programme.	- Programme was integrated into the School Anaemia Control Programme (SACP) which is a specific platform for delivering health and nutrition services for school-aged children and adolescents.	

			- Birth weight (child).			- SACP was created in collaboration with the Department of Health and Family Welfare.<br>- SACP provides adolescent girls (aged 14-17 years) attending school with one IFA supplement per week and deworming tablets twice a year.	
	Roche et al., 2018 [61]	Indonesia	- Anaemia (maternal).	- Adolescent girls.<br>- WRA.	- IFA supplementation programme.	- Indonesia's Ministry of Health amended its national programme for prevention of anaemia in WRA and adolescent girls to align with WHO guidance and the programme is delivered through the existing school health programme (names UKS/M).	
	Soekarjo et al., 2018 [70]	Indonesia	- Anaemia (maternal).	- Adolescent girls.	- IFA supplementation programme.	- There is only one nutrition-specific policy and programme that is designed to improve adolescent nutritional status; the IFA supplementation programme has been integrated into the Guideline for the Prevention and Management of	- There is a lack of policy and programmes to improve adolescent girls' nutritional status and the existing programmes are yet to be scaled up.

						Anaemia among Adolescent Girls and WRA.	
	Wadhwa et al., 2018 [81]	India	- Anaemia (maternal).	- Adolescent girls.	- IFA supplementation programme.	- Programme has been integrated into adolescent-friendly health clinics.<br>- National Adolescent Reproductive and Sexual Health Policy (2006) involves nutrition-specific policies for adolescent girls.	
Antenatal/Postnatal clinics							
	Ajayi et al., 2013 [23]	Nigeria	- Mortality (maternal).<br>- Morbidity (maternal).	- Pregnant women.	- ANC services.	- State Ministry of Health and Federal Ministry of Health established guidelines and policy on health interventions; this included national level policy on nutrition programmes targeting pregnant women.<br>- ANC services were delivered through primary health facilities.<br>- The Primary Health for Maternal Care carried out a maternal mortality and morbidity reduction intervention where volunteers identified	

						pregnant women and encouraged them to attend ANC clinics.	
	Amoah et al., 2016 [18]	Ghana	- Morbidity (maternal).	- Pregnant women.	- Mobile health service.	- Programme was delivered via CHWs and encouraged ANC attendance.<br>- CHWs were given mobile phones and would input pregnancy data given by mothers; mothers would then report maternal indicators (such as bleeding, abdominal pain, etc.) and CHWs would be instructed on what to advise the pregnant women and would then link them with ANC services.	
	Appiah et al., 2016 [33]	Ghana	- Anaemia (maternal).<br>- Knowledge of anaemia (maternal).<br>- Adherence to anaemia prevention strategies (maternal).	- Pregnant women.	- Anaemia prevention via ANC services.	- Programme was integrated into ANC services and into 25 health facilities including a hospital, 12 community-based health planning and services compounds, 5 clinics and 7 maternity homes within the community.	
	Arega Sadore et al., 2015 [36]	Ethiopia	- Adherence to taking IFA supplements (maternal).<br>- Knowledge of	- Pregnant women.	- IFA supplementation programme.	- Programme was integrated into ANC services and into seven health centres and	- Author concluded that health workers promoting mothers to attend ANC at least 4 times could improve compliance to IFA

			anaemia (maternal).			thirty five health posts within the community.	supplementation programmes; more effort needs to be directed towards mobilising community health workers in this area to improve maternal health outcomes.
	Babughirana et al., 2020 [38]	Uganda	- Mortality (maternal).<br>- Adherence to taking IFA supplements (maternal).<br>- Breastfeeding practices (maternal).	- WRA who had a child aged 0-59 months.	- ANC services with IFA supplementation programme.	- Ugandan Ministry of Health have set targets to reduce maternal and newborn mortality as part of their Uganda maternal newborn and child health sharpened plan; every 5 years the government of Uganda commission a health and demographic survey to gauge uptake of services including ANC services.	- Author concluded that ANC service uptake was poor and the quality of care in ANC services across the region was also poor; this region has the highest maternal mortality rate in Uganda, this is linked to poor neonatal and maternal care services across this region.
	Barker et al., 2019 [11]	Sub-Saharan Africa	- Breastfeeding practices (maternal).<br>- Undernourishment (maternal).<br>- Undernourishment (child).	- Pregnant women living with HIV.	- Nutrition counselling (including breastfeeding practices), Iodised salt fortification programme, and Folic acid fortification programme.	- Programme was scaled-up and integrated into perinatal and antenatal services as part of the Partnership for HIV-free Survival initiative, with the assistance of Ministry of Health departments across Tanzania and Uganda.<br>- Kenya, Mozambique and Lesotho delivered the programme, however there was less engagement and no plans to scale-up.	- Further integration is needed into government programmes and better sustainability of quality insurance capability throughout health systems.

	Berti et al., 2018 [12]	Central America, Southeast Asia, South Asia, Sub-Saharan Africa	- Anaemia (maternal).<br>- Iron status (maternal).<br>- Neonatal outcomes (child).	- Pregnant women.<br>- WRA.	- Multiple micronutrient supplementation programme.	- Nepal: implementation of IFA supplementation as part of the Iron Intensification Project within Nepal's National Plan for Action for the Control of Anaemia. Government of Nepal have strong partnerships with NGOs in supporting IFA supplementation integration into ANC.	- Implementation and integration strategies were most effective in programmes that were facilitated by continued promotional and education activities, and that were context-specific.
	Bucher et al., 2015 [14]	Argentina, Guatemala, India, Kenya, Pakistan, Zambia	- Gestational weight (maternal).<br>- Haemoglobin level (maternal).<br>- Prenatal vitamin/iron consumption (maternal).	- Mothers.	- ANC services and anaemia screening.	- ANC services were integrated and delivered as part of a government hospital, private hospital, government clinic, private clinic, or via a health worker.<br>- Government hospitals were the most commonly reported location where women attended ANC.	- Coverage for prenatal vitamins/iron delivery was above 90% in India, Zambia, Kenya and Guatemala and above 73% in Argentina and Pakistan.
	De Silva et al., 2019 [4]	Asia and the Pacific	- Birth weight (child).<br>- Energy deficiency (maternal).	- Women of reproductive age (WRA).	- Energy & protein supplementation programme and IFA supplementation programme.	- Supplementation policies have been implemented and aimed at reducing undernutrition and micronutrient deficiencies amongst WRA.<br>- 9 out of 11 countries in the South-East region and 15 out of 26 countries in the	- Advocates for expanding nutrition policies, integrating surveillance data and implementing targeted interventions to improve adolescent health and nutrition outcomes.

						Western Pacific region had incorporated goals/targets relating to maternal nutrition services into their national policies.	
	Ejigu et al., 2013 [22]	Ethiopia	- Anaemia (maternal).<br>- Received IFA supplements (maternal).	- Pregnant women.	- ANC services and IFA supplementation.	- ANC services were integrated into eight public health facilities.	
	Gebremichael et al., 2019 [27]	Ethiopia	- Adherence to taking IFA supplements (maternal).<br>- Knowledge of anaemia (maternal).	- Pregnant women.<br>- WRA.	- IFA supplementation programme.	- Programme was integrated into ANC services and delivered in a hospital.	
	Ghosh-Jerath et al., 2015 [29]	India	- Anaemia (maternal).<br>- Protein intake (maternal).<br>- Micronutrient intake of iron, calcium, vitamin A, vitamin C, thiamine, riboflavin, niacin, zinc and vitamin B12 (maternal).	- Pregnant women.<br>- Mothers.	- ANC services with nutrition education and counselling.	- Nutrition education and counselling was integrated into ANC services in Delhi, India.<br>- Pregnant women involved in this study all had access to a healthcare facility (government or private) that specialised in MNCH care within a 2.5km radius of where they lived.<br>- 17 private MNCH clinics, one government-run MNCH hospital and several referral hospitals were also situated in the area that	

						delivered ANC services.	
	Gilder et al., 2019 [30]	Myanmar, Thailand	- Anaemia (maternal).	- Pregnant women.<br>- Mothers.	- ANC services and anaemia screening/treatment.	- ANC services were integrated into rural health clinics which offer free healthcare and ANC to refugee and migrants from Myanmar.	
	Izudi et al., 2017 [31]	Uganda	- Acute malnutrition (MUAC) (maternal).	- Pregnant women.	- ANC services and malnutrition screening using MUAC.	- ANC services were integrated into Kaabong hospital which is owned by the government of the republic of Uganda.	- Author highlights the importance of quality improvement in nutritional assessment, counselling and support at all service delivery points within the healthcare system.
	Kavle et al., 2019 [91]	Malawi	- Breastfeeding practices (maternal).	- Pregnant women.<br>- Mothers.	- Baby-friendly hospital initiative (BFHI) (equipping healthcare providers with skills on breastfeeding counselling & early initiation).	- BFHI was scaled-up and integrated in health areas under the Maternal and Child Survival Programme (supported by the Government of Malawi and Ministry of Health), (this included any hospital or health facility with a ANC or maternity ward).<br>- Engagement of local leadership at both community, district and national levels was crucial in effective scale-up and integration.	- Full integration of this programme required investing in training for healthcare workers, improved monitoring and evaluation systems and a greater push for breastfeeding support at the community level.

	Kim et al., 2017 [37]	India	- Neonatal mortality (child).<br>- Mortality (maternal).	- WRA.<br>- Pregnant women.	- ANC care services.	- Programme was delivered through two government services: the Integrated Child Development services and National Rural Health Mission in India.<br>- ANC services were introduced as part of this programme.	- Though the programme is delivered through government health services, there is a lack of convergence with health and nutrition policy at state level.
	Kiwanuka et al., 2017 [39]	Uganda	- Adherence to taking IFA supplements (maternal).	- Pregnant women.	- Iron supplementation programme.	- Programme was integrated into ANC services and delivered in a hospital.	
	Lassi et al., 2013 [40]	Global	- Neonatal mortality (child).<br>- Small for gestational age (child).<br>- Stillbirth (child).<br>- Birth weight (child).	- WRA.	- Energy & protein supplementation programme.	- Programme was delivered through ANC services to WRA.	- There is a significant lack of research on the effect of energy and protein supplementation on both child and maternal outcomes.
	Lyngdoh et al., 2018 [43]	India	- Adherence to taking IFA supplements (maternal).	- WRA.<br>- Mothers.	- ANC services with IFA supplementation programme.	- Programme was delivered through ANC services as part of the Uttar Pradesh Community Mobilization project whose aim was the improve RMNCH behaviours.	

	Mgamb et al., 2017 [47]	Kenya	- Folate deficiency (maternal).<br>- Knowledge of folic acid fortified flour (maternal).	- Pregnant women.	- Folic acid fortified flour programme.	- Programme was integrated into ANC services at a maternity hospital.<br>- Government of Kenya made fortification of maize and wheat flour with folic acid mandatory in 2012.	
	Mistry et al., 2018 [48]	India	- Anaemia (maternal).<br>- Iron-deficiency anaemia (maternal).	- Pregnant women.	- Iron supplementation programme.	- Programme was integrated into ANC services in government health centres.	- Good systems in place for tackling anaemia however policy-makers are interested in integrating antenatal tobacco screening for anaemia control into these services.
	Nguyen et al., 2021 [51]	India	- Breastfeeding practices (maternal).<br>- Anthropometric measurements (child).	- WRA.	- ANC services.	- The Anaemia Mukt Bharat programme set targets to reduce anaemia prevalence amongst pregnant women - part of the programme strategy involved supplying IFA supplements and providing ANC services.	
	Noznesky et al., 2012 [54]	India	- Birth weight (child).<br>- Energy deficiency (maternal).<br>- Underweight (maternal).<br>- Anaemia (maternal).<br>- Birth weight (child).	- WRA.	- IFA supplementation, Energy-protein supplementation and Nutrition education programmes.	- Programme was delivered as part of the government of Bihar's strategy to reduce maternal undernutrition by implementing several interventions aimed at improving maternal and birth outcomes, through ANC clinics.	- Advocates for increased support and supervision of CHWs to improve training, problem-solving abilities and flow of information.

	Ouedraogo et al., 2019 [56]	Niger	- Adherence to taking IFA supplements (maternal).<br>- Gestational weight (maternal).<br>- Anaemia (maternal).	- Pregnant women.	- IFA supplementation programme.	- Programme was integrated into ANC services and selected integrated health centres.	
	Phillips et al., 2017 [58]	Haiti	- Hypertension (maternal).<br>- Knowledge of breastfeeding practices (maternal).<br>- Knowledge of obstetric complications (maternal).	- Pregnant women.	- ANC mobile clinics with IFA supplementation.	- 130 ANC mobile clinics were rolled out as part of the national MCHN programme funded by USAID in Haiti.	
	Saldanha et al., 2012 [65]	Ethiopia	- Anaemia (maternal).<br>- Thinness (maternal).<br>- Stunting (maternal).	- Mothers.<br>- Pregnant and lactating women.	- Nutrition education and counselling programme and IFA supplementation programme.	- Programme was integrated into ANC services and community health workers were employed to mobilise women to attend ANC.<br>- Health extension programme was scaled-up to improve pregnant and lactating women in their access to health services.	

	Thapa et al., 2016 [73]	Nepal	- Pre-eclampsia (maternal).<br>- Gestational age (child).<br>- Adherence to taking calcium supplements (maternal).	- Pregnant women.	- Calcium supplementation and counselling programme.	- Programme was integrated into ANC services and delivered via CHWs.<br>- CHWs in the form of Female community health volunteers were trained to distribute calcium supplements and counsel pregnant women about calcium use by reinforcing calcium-related health messages.	
	Torlesse et al., 2021 [74]	Afghanistan, Bangladesh, Nepal, India, Pakistan	- Meal frequency (maternal).<br>- Adherence to taking IFA and multiple micronutrient supplements (maternal).<br>- Consumption of nutritious and diverse foods (maternal).<br>- Quantity of food intake (maternal).	- WRA.<br>- Pregnant and lactating women.	- Nutrition education and counselling (including IYCF practices).	- Programme was integrated into ANC services and counselling was provided at health facility, community, household and individual levels.	- Author states that inequalities in access to nutrition services as well as limited investment into training for health workers constrains the quality of counselling.<br>- More needs to be done to integrate counselling into M&E mechanisms in order to assess where improvements are needed.
	Young et al., 2018 [84]	Kenya	- Anaemia (maternal).	- Pregnant women.	- ANC services.	- ANC services were integrated into 37 public health facilities: 1 district hospital, 9 health centres and 27 dispensaries (dispensaries make up the lowest level of the formal healthcare system, offering basic maternal and child	

						healthcare and receive around 40 ANC visits monthly).	
Family planning services							
	Kavle et al., 2017 [92]	Egypt	- Stunting (child).	- Pregnant women.<br>- Mothers with children under 2 years (lactating and non-lactating).	- Family planning services (including advice on exclusive breastfeeding and birth spacing).	- This service was multi-faceted and involved a combination of different strategies to improve growth and development of child (including covering breastfeeding, complementary feeding, prevention of infection, household and family factors, maternal diet, etc.)	- Counselling within Family planning services and Maternal, infant and young child nutrition (MIYCN) services should be strengthened in order to improve quality across antenatal and postnatal platforms.
	Kavle et al., 2019 [91]	Malawi	- Breastfeeding practices (maternal).	- Pregnant women.<br>- Mothers.	- Baby-friendly hospital initiative (BFHI) (equipping healthcare providers with skills on breastfeeding counselling & early initiation).	- BFHI was scaled-up and integrated in health areas under the Maternal and Child Survival Programme (supported by the Government of Malawi and Ministry of Health), including within Family Planning services at both the health facility and community level (this also included any hospital or health facility with a maternity ward).<br>- Engagement of local leadership at both	- Full integration of this programme required investing in training for healthcare workers, improved monitoring and evaluation systems and a greater push for breastfeeding support at the community level.

						community, district and national levels was crucial in effective scale-up and integration.	
	Mason et al., 2012 [45]	Ethiopia, India, Nigeria, Global	- Birth weight (child).<br>- Neonatal growth (child).<br>- Morbidity (maternal).<br>- Mortality (maternal).	- WRA.<br>- Pregnant and lactating women.	- Family planning services (including advice on birth spacing).	- The Bihar National Rural Health Mission launched the Healthy Timing and Spacing of Pregnancies initiative which was delivered through Family planning services in India.<br>- In Ethiopia, government campaigns are being carried out to reduce maternal mortality and promote the use of Family planning services for birth spacing advice.<br>- Family planning services in Nigeria were available through health facilities.	- Awareness of the importance of maternal nutrition was observed as a limiting factors when identifying how well integrated maternal nutrition services were.<br>- There is adequate support and funding for maternal nutrition services and is often low priority; author calls for a information campaign to raise awareness of its importance.
	Noznesky et al., 2012 [54]	India	- Birth weight (child).<br>- Energy deficiency (maternal).<br>- Underweight (maternal).<br>- Anaemia (maternal).<br>- Birth weight (child).	- WRA.<br>- Adolescent girls in school.	- Family planning services.	- Family planning services are provided to women through the primary health system, with CHWs promoting these services.<br>- Cash incentives are offered to WRA, CHWs and service providers.	- Currently there is low utilisation of Family planning services in Bihar.<br>- Laws to prohibit child marriage and encourage adolescent girls to stay in school and wait until they are 18 to get married could be more effective in preventing teenage pregnancies than Family planning services.

	Varghese et al., 2014 [77]	India	- Neonatal mortality (child).<br>- Breastfeeding initiation within 5 hours postpartum (maternal).	- Pregnant and lactating women.<br>- Mothers.	- Family planning services.	- Programme was launched as part of a plan to improve maternal and neonatal care and integrated into various health facilities (mainly hospitals) across India.<br>- Health workers, called "Yashodas" were places at hospitals and health facilities to provide support and care for mothers during their stay; this included nutrition counselling, breastfeeding counselling and family planning advice.	- Scaling up this programme is recommended in district hospitals and other health facilities that have a higher number of births.
Reproductive health services							
	Tappis et al., 2020 [72]	Yemen	- Mortality (child).<br>- Mortality (maternal).<br>- Morbidity (child).<br>- Morbidity (maternal).	- WRA.	- Reproductive, Maternal, Newborn and Child health services (RMNCH) including IFA supplementation, antenatal and postnatal care.	- Maternal health and nutrition service delivery was assessed across 3 of Yemen's 22 governates (in collaboration with Ministry of Public Health and Population).<br>- 16 facility-based healthcare providers were responsible for the delivery of RMNCH services across.<br>- Majority of services provided through reproductive health platforms are delivered	- Advocates for a comprehensive package delivery of RMNCH services through investing CHW training and decentralisation of health services.

						by CHWs (female health workers).	
	Wadhwa et al., 2018 [81]	India	- Anaemia (maternal).	- Adolescent girls.	- IFA supplementation programme.	- National Adolescent Reproductive and Sexual Health Policy (2006) involves nutrition-specific policies for adolescent girls.<br>- Programme has been integrated into adolescent-friendly health clinics as a result of above policy and the Reproductive, Maternal, Newborn, Child and Adolescent Health strategy (2013).	

Supplementary Table S4 : Summary of literature findings by delivery platform

Delivery platforms	Number of studies	Examples of integration	Key recommendations	Barriers to integration
CHW programmes	*n=7*<br><br>Altobelli, et al. 2017.<br>Amoah, et al. 2016.<br>Noznesky, et al. 2012.<br>Ruton, et al. 2018.<br>Salam, et al. 2016.<br>Stansert Katzen, et al. 2020.<br>Thapa, et al. 2016.	ANC services, Primary Healthcare System, Community Healthcare Centres, School Anaemia Program,	Expand policies and guidelines to support CHWs; invest in training, support and supervision of CHWs	Lack of training, lack of support and poor mechanisms for large-scale supervision of CHWs
ANC services	*n=29*<br><br>Ajayi, et al. 2013.<br>Amoah, et al. 2016.<br>Appaih, et al. 2016.<br>Arega Sadore, et al. 2015.<br>Babughirana, et al. 2020.<br>Barker, et al. 2019.<br>Berti, et al. 2018.<br>Bucher, et al. 2015.<br>De Silva, et al. 2019.<br>Ejigu, et al. 2013.<br>Gebremichael, et al. 2019.<br>Ghosh-Jerath, et al. 2015.<br>Gilder, et al. 2019.<br>Izudi, et al. 2017.<br>Kavle, et al. 2019.<br>Kim, et al. 2017.<br>Kiwanuka, et al. 2017.	Primary Healthcare System, Community Healthcare Centres, Maternal and Child Survival Program, Integrated Child Development Services,	Mobilise CHWs to increase awareness of the importance of attending ANC appointments; improve quality of care amongst ANC services; implement promotional and education campaigns to target GWRA	Lack of coordination amongst Ministry of Health directorates, lack of awareness around the importance of women’s health

	Lassi, et al. 2013.<br>Lyngdoh, et al. 2018.<br>Mgamb, et al. 2017.<br>Mistry, et al. 2018.<br>Nguyen, et al. 2021.<br>Noznesky, et al. 2012.<br>Ouedraogo, et al. 2019.<br>Phillips, et al. 2017.<br>Saldanha, et al. 2012.<br>Thapa, et al. 2016.<br>Torlesse, et al. 2021.<br>Young, et al. 2018.			
Family planning services	*n=4*<br><br>Kavle, et al. 2018.<br>Kavle, et al. 2019.<br>Noznesky, et al. 2012.<br>Varghese, et al. 2014.	Maternal and Child Survival Program, Primary Healthcare System, Baby-friendly Hospital Initiative	Integrate nutrition counselling into family planning services; implement information campaigns to raise awareness and create demand for services; scale up this service in health facilities that have a higher number of births; strengthen policies and programs to postpone teenage pregnancy	Lack of awareness around the importance of women's health
Reproductive health services	*n=1*<br><br>Tappis, et al. 2020.	Reproductive, Maternal, Newborn and Child Health Services, Primary Healthcare System, National Policy, Adolescent-friendly Health Clinics	Implement a comprehensive package delivery of Reproductive, Maternal, Newborn and Child Health Services; target non-pregnant, non-lactating adolescents or GWRA; utilise schools as a delivery platform; implement information campaigns to raise awareness and create demand for services;	Lack of awareness around the importance of adolescent health
Adolescent health services	*n=4*<br><br>Noznesky, et al. 2012.<br>Roche, et al. 2018.<br>Soekarjo, et al. 2018.	School Anaemia Control Programme, Indonesia's Ministry of Health national programme for prevention of	Target non-pregnant, non-lactating adolescents or GWRA; utilise schools as a delivery platform; implement information campaigns to raise	Lack of awareness around the importance of adolescent health

	Wadhwa, et al. 2018	anaemia in WRA, School Health Programme	awareness and create demand for services;	

PRISMA 2020 Checklist

Supplementary Table S5: PRISMA checklist

Section and Topic	Item #	Checklist item	Location where item is reported
**TITLE**			
Title	1	Identify the report as a systematic review.	1, 2
**ABSTRACT**			
Abstract	2	See the PRISMA 2020 for Abstracts checklist.	1
**INTRODUCTION**			
Rationale	3	Describe the rationale for the review in the context of existing knowledge.	1, 2
Objectives	4	Provide an explicit statement of the objective(s) or question(s) the review addresses.	2
**METHODS**			
Eligibility criteria	5	Specify the inclusion and exclusion criteria for the review and how studies were grouped for the syntheses.	3
Information sources	6	Specify all databases, registers, websites, organisations, reference lists and other sources searched or consulted to identify studies. Specify the date when each source was last searched or consulted.	2, 3
Search strategy	7	Present the full search strategies for all databases, registers and websites, including any filters and limits used.	Supplementary table 1 and 2
Selection process	8	Specify the methods used to decide whether a study met the inclusion criteria of the review, including how many reviewers screened each record and each report retrieved, whether they worked independently, and if applicable, details of automation tools used in the process.	3, 4
Data collection process	9	Specify the methods used to collect data from reports, including how many reviewers collected data from each report, whether they worked independently, any processes for obtaining or confirming data from study investigators, and if applicable, details of automation tools used in the process.	3, 4
Data items	10a	List and define all outcomes for which data were sought. Specify whether all results that were compatible with each outcome domain in each study were sought (e.g. for all measures, time points, analyses), and if not, the methods used to decide which results to collect.	3, 4
	10b	List and define all other variables for which data were sought (e.g. participant and intervention characteristics, funding sources). Describe any assumptions made about any missing or unclear information.	3, 4, supplementary table 2
Study risk of bias assessment	11	Specify the methods used to assess risk of bias in the included studies, including details of the tool(s) used, how many reviewers assessed each study and whether they worked independently, and if applicable, details of automation tools used in the process.	N/A
Effect measures	12	Specify for each outcome the effect measure(s) (e.g. risk ratio, mean difference) used in the synthesis or presentation of results.	3
Synthesis methods	13a	Describe the processes used to decide which studies were eligible for each synthesis (e.g. tabulating the study intervention characteristics and comparing against the planned groups for each synthesis (item #5)).	3, 4,
	13b	Describe any methods required to prepare the data for presentation or synthesis, such as handling of missing summary statistics, or data conversions.	3, 4
	13c	Describe any methods used to tabulate or visually display results of individual studies and syntheses.	3, 4
	13d	Describe any methods used to synthesize results and provide a rationale for the choice(s). If meta-analysis was performed, describe the model(s), method(s) to identify the presence and extent of statistical heterogeneity, and software package(s) used.	3, 4
	13e	Describe any methods used to explore possible causes of heterogeneity among study results (e.g. subgroup analysis, meta-regression).	3, 4
	13f	Describe any sensitivity analyses conducted to assess robustness of the synthesized results.	3, 4

PRISMA 2020 Checklist

Section and Topic	Item #	Checklist item	Location where item is reported
Reporting bias assessment	14	Describe any methods used to assess risk of bias due to missing results in a synthesis (arising from reporting biases).	N/A
Certainty assessment	15	Describe any methods used to assess certainty (or confidence) in the body of evidence for an outcome.	3, 4
**RESULTS**			
Study selection	16a	Describe the results of the search and selection process, from the number of records identified in the search to the number of studies included in the review, ideally using a flow diagram.	4
	16b	Cite studies that might appear to meet the inclusion criteria, but which were excluded, and explain why they were excluded.	4
Study characteristics	17	Cite each included study and present its characteristics.	Supplementary table 3
Risk of bias in studies	18	Present assessments of risk of bias for each included study.	N/A
Results of individual studies	19	For all outcomes, present, for each study: (a) summary statistics for each group (where appropriate) and (b) an effect estimate and its precision (e.g. confidence/credible interval), ideally using structured tables or plots.	5, 6, 7, 8
Results of syntheses	20a	For each synthesis, briefly summarise the characteristics and risk of bias among contributing studies.	N/A
	20b	Present results of all statistical syntheses conducted. If meta-analysis was done, present for each the summary estimate and its precision (e.g. confidence/credible interval) and measures of statistical heterogeneity. If comparing groups, describe the direction of the effect.	7, 8
	20c	Present results of all investigations of possible causes of heterogeneity among study results.	7, 8
	20d	Present results of all sensitivity analyses conducted to assess the robustness of the synthesized results.	7, 8
Reporting biases	21	Present assessments of risk of bias due to missing results (arising from reporting biases) for each synthesis assessed.	N/A
Certainty of evidence	22	Present assessments of certainty (or confidence) in the body of evidence for each outcome assessed.	7, 8
**DISCUSSION**			
Discussion	23a	Provide a general interpretation of the results in the context of other evidence.	9, 10, 11
	23b	Discuss any limitations of the evidence included in the review.	11
	23c	Discuss any limitations of the review processes used.	11
	23d	Discuss implications of the results for practice, policy, and future research.	11
**OTHER INFORMATION**			
Registration and protocol	24a	Provide registration information for the review, including register name and registration number, or state that the review was not registered.	3
	24b	Indicate where the review protocol can be accessed, or state that a protocol was not prepared.	3
	24c	Describe and explain any amendments to information provided at registration or in the protocol.	3
Support	25	Describe sources of financial or non-financial support for the review, and the role of the funders or sponsors in the review.	Title page
Competing interests	26	Declare any competing interests of review authors.	Title page
Availability of data, code and	27	Report which of the following are publicly available and where they can be found: template data collection forms; data extracted from included studies; data used for all analyses; analytic code; any other materials used in the review.	Title page

PRISMA 2020 Checklist

Section and Topic	Item #	Checklist item	Location where item is reported
other materials			

From: Page MJ, McKenzie JE, Bossuyt PM, Boutron I, Hoffmann TC, Mulrow CD, et al. The PRISMA 2020 statement: an updated guideline for reporting systematic reviews. BMJ 2021;372:n71. doi: 10.1136/bmj.n71

For more information, visit: http://www.prisma-statement.org/
